# James O'Hara Scanlon

**Published:** 1911-08-19

**Authors:** 


James O'Hara Scanlon, Lieutenant-Colonel of the retired Army Medical Staff, has died at Broadclyst, Devon, at the age of sixty-four. He was a graduate of the University of Glasgow, and took degrees of M.B. and C.M. in 1868.

